# Evaluation of the protective effect of curcumin on encephalopathy caused by intrahepatic and extrahepatic damage in male rats

**DOI:** 10.22038/ijbms.2021.53171.11976

**Published:** 2021-06

**Authors:** Forouzan Frozandeh, Nader Shahrokhi, Mohammad Khaksari, Sedigheh Amiresmaili, Gholamreza AsadiKaram, Nava Shahrokhi, Maryam Iranpour

**Affiliations:** 1 Physiology Research Center, Institute of Neuropharmacology, Kerman University of Medical Sciences, Kerman, Iran; 2 Neuroscience Research Center, Institute of Neuropharmacology, Kerman University of Medical Sciences, Kerman, Iran; 3 Endocrinology and Metabolism Research Center, Institute of Basic and Clinical Physiology Sciences, Kerman University of Medical Sciences, Kerman, Iran; 4 Department of Physiology, Bam University of Medical Sciences, Bam, Iran; 5 Medical School, Kerman University of Medical Sciences, Kerman, Iran; 6 Pathology and Stem Cell Research Center, Kerman University of Medical Sciences, Kerman, Iran

**Keywords:** Acetaminophen, Brain edema, Curcumin, Hepatic damage, Hepatic encephalopathy, Intracranial pressure

## Abstract

**Objective(s):**

Along with increased intracranial pressure (ICP) and brain damage, brain edema is the most common cause of death in patients with hepatic encephalopathy. Curcumin can pass the blood-brain barrier and possesses anti-inflammatory and anti-oxidant properties. This study focuses on the curcumin protective effect on intrahepatic and extrahepatic damage in the brain.

**Materials and Methods:**

One hundred and forty-four male Albino N-Mary rats were randomly divided into 2 main groups: intrahepatic injury group and extrahepatic cholestasis group. In intra-hepatic injury group intrahepatic damage was induced by intraperitoneal (IP) injection of acetaminophen (500 mg/kg) [19] and included four subgroups: 1. Sham, 2. Acetaminophen (APAP), 3. Normal saline (Veh) which was used as curcumin solvent, and 4. Curcumin (CMN). In extrahepatic cholestasis group intrahepatic damage was caused by common bile duct litigation (BDL) and included four subgroups: 1. Sham, 2. BDL, 3. Vehicle (Veh), and 4. Curcumin (CMN). In both groups, 72 hr after induction of cholestasis, brain water content, blood-brain barrier permeability, serum ammonia, and histopathological indicators were examined and ICP was measured every 24 hr for three days.

**Results:**

The results showed that curcumin reduced brain edema, ICP, serum ammonia, and blood-brain barrier permeability after extrahepatic and intrahepatic damage. The maximum effect of curcumin on ICP was observed 72 hr after the injection.

**Conclusion:**

According to our findings, it seems that curcumin is an effective therapeutic intervention for treating encephalopathy caused by extrahepatic and intrahepatic damage.

## Introduction

Hepatic encephalopathy (HE) is a neuropsychological disorder in patients with acute or chronic liver failure (1) and liver failure is caused by extrahepatic and intrahepatic damages. Cholestasis leads to liver cell death, fibrosis, cirrhosis, and ultimately, liver failure (2). In cholestasis, production, secretion, and bile flow are associated with functional impairment (3). Besides, accumulation of some substances in the blood stimulates pro-inflammatory cytokines and increases apoptosis in liver cells (4). Studies have shown that in cholestasis, the liver antioxidant capacity drops (5). 

TAccumulation of hydrophobic bile acids in the blood plays a role in the induction of apoptosis, necrosis, and increased oxidative stress (6). The level of vascular permeability-enhancing substances (bile acids, substance P, and histamine) increases in cholestasis (7), and the resulting toxicity of bile acids in the brain during cholestasis can be one of the causes of HE (2). Furthermore, acetaminophen overdose is known as one of the causes of intrahepatic damage (8). 

Acetaminophen can cause liver damage through several mechanisms including increased oxidative stress, altered calcium homeostasis, immediate mitochondrial permeability, removal of mitochondrial membrane potential, ability of mitochondria to make ATP, and necrosis (9).

Encephalopathy is defined as changes in the physical condition, cognitive function, and circadian rhythm. It includes a range of clinical signs such as deficits in attention, motivation, memory, and learning, losing consciousness, restlessness, depression or euphoria, drowsiness, forgetfulness, irritability, delirium, convulsions, and deep coma (10). Brain edema, along with increased ICP and brain herniation, is the most common cause of death in patients with HE (11).

HHE is characterized by morphological changes in astroglia cells.

These changes affect the patients’ quality of life and their ability to perform activities of daily living (12). Thus, prevention and treatment can play an important role in improving human health and quality of life. 

Curcumin ([1,7-bis-(4-hydroxy-3-methoxyphenyl)-1,6-heptadiene-3,5-dione) is the biologically active compound of turmeric (a perennial herbaceous plant and a Zingiberaceae family member) that grows in the eastern regions of Asia. Most of the health benefits of turmeric including antioxidant, anti-inflammatory, anti-bacterial, and hepatoprotective effects are related to curcumin [1,7-bis-(4-hydroxy-3-methoxyphenyl)-1,6-heptadiene-3,5-dione](13, 14).

Since curcumin passes the blood-brain barrier (BBB), it is used for degenerative neurological disorders (15). This study aimed to investigate the potential healing effects of curcumin on the BBB permeability, ICP, brain edema, plasma ammonia levels, and pathological changes in encephalopathy caused by bile duct obstruction and acetaminophen overdose.

## Materials and Methods

This interventional-experimental study was conducted in Afzalipour Medical School, Kerman University of Medical Sciences. One hundred and forty-four adult male Wistar rats (200 - 220 g) were kept at a temperature of 20 to 22 °C and a light-dark cycle of 12 hr in the Medical School’s animal house. The humidity was 40%-60%, and food and water were available ad libitum. This study was done at Kerman University of Medical Sciences under the permission of the Research Ethics Committee IR with license number IR.kmu.REC.1394,542. 

***Drugs***

Curcumin was purchased from Santa Cruz Company, USA. Kerman University of Medical Sciences, Faculty of Pharmacy provided the acetaminophen. 

***Animal grouping***

The animals were randomly divided into two main groups: Intrahepatic injury and extrahepatic cholestasis. A: In intra-hepatic injury group intrahepatic damage was induced by IP injection of acetaminophen (500 mg/kg)(16) and included 4 subgroups: 1. Sham, 2. Acetaminophen, (APAP) 3. Normal saline (Veh) which was used as curcumin solvent, 4. Curcumin (CMN). B: In extrahepatic cholestasis group intrahepatic damage was caused by common bile duct litigation (BDL) and included 4 subgroups: 1. Sham, 2. BDL, 3. Vehicle (Veh), and 4. Curcumin (CMN). Curcumin (300 mg/kg) [20] was administered (IP) 24 hr after the injection of acetaminophen in the first group and 28 days following BDL in the second group (17).

***Extrahepatic cholestasis induction***

 The animals were anesthetized by IP ketamine (50 mg/kg (and xylazine (10 mg/kg). The abdominal layers were cut carefully to observe the abdominal viscera. The main bile duct was first ligated using two ligatures approximately 0.5 cm apart and then transected at the midpoint between the two ligatures to maintain sterile conditions. The abdominal wall was sutured in layers of fascia using 4-0 absorbable sutures, and the skin was stitched using 4-0 non-absorbable sutures (18).

***Intrahepatic damage induction***

 The animals were kept without food 12 hr before damage induction and had free access to water. The intrahepatic damage was induced by an IP single injection of acetaminophen (500 mg/kg) (16).

***Measurement of plasma ammonia levels***

Blood samples were taken from the abdominal aorta, and plasma ammonia levels were measured using a UV kit and enzymatic method at the end of the treatment period.

***Determination of BBB permeability***

At the end of the treatment period rats were injected with 20 mg/kg (2%) of Evans blue (EB). One hour later, the animals’ thorax was opened under deep anesthesia. A needle was inserted into the left ventricle and rinsed with 200–300 ml of isotonic saline solution for 20 min until clear solution exited through the external jugular vein. Then, the brain was immediately removed and placed in a 20-ml solution containing acetone and sodium sulfate and shaken for 24 hr. The resulting supernatant was used to measure the EB absorbance at 620 nm wavelength using a spectrophotometer (Biotech Pharmacia, Munich, Germany). The following formula was used to calculate the Evans blue dye content (19).

Evans blue content (µg/mg tissue)= (13.24×20×absorbance) / tissue weight

***Determination of brain edema***

At the end of the treatment period, brain edema was determined by measuring brain water content as follows: the whole brain was quickly removed under anesthesia, and it was weighed (wet weight). Then it was kept at 60–70 °C for 72 hr and reweighed (dry tissue weight). Finally, using the following formula, the amount of water was calculated as an index of edema (20):

Brain water content %= ([wet weight - dry weight] / wet weight) × 100

***ICP measurement***

 ICP was measured using a premade ICP monitoring system. After anesthesia with ketamine (80 mg/kg) and xylazine (10 mg/kg), the animals were put in a stereotaxic apparatus. After determining the occipital bone projection by touching its lower end, polyethylene tube No. 10 (PE10) was slowly inserted into the cisterna magna space. Then, the system was connected to a pressure transducer through a stopcock, and the pressure was recorded by a computer (21). ICP in the intra-hepatic injury group was done 24 hr after administration of acetaminophen at different times 0, 24, 48, and 72 hr after intrahepatic damage. Also, ICP in the extrahepatic cholestasis group was done 28 days after BDL at different times: 0, 24, 48, and 72 hr after extrahepatic injury.

 ***Histopathology ***

The animal’s brain tissue was placed in 10% formalin-fixed paraffin, and after staining with Hematoxylin and Eosin, the tissue was examined. Tissue sections were analyzed regarding their morphological changes. In both intrahepatic and extrahepatic cholestasis, brain tissue edema was analyzed, categorized into three groups: mild, moderate, and severe. This biomarker was investigated 28 days after BDL of brain tissue in the extrahepatic cholestasis group (22).

***Statistical methods***

 Results are presented as the mean ± SEM. One-way analysis of variance (ANOVA) was performed to determine the differences among the groups followed by Tukey’s *post-hoc* test for differences between groups. *P*<0.05 was considered statistically significant.

## Results

***Brain water content***

 The amount of brain water in different groups, 72 hr after extrahepatic and intrahepatic cholestasis, is shown in Figure 1. Figure 1-A shows a significant increase in brain water content in rats that received acetaminophen (APAP) compared with the sham group (82.01 ± 0.35) (*P*<0.001). The brain water content decreased significantly in APAP + CMN (79.25 ± 0.17) compared with APAP and curcumin vehicles. Figure 1-B shows that brain water content in the BDL group (79.22 ± 0.20) had a significant increase compared with the sham group (*P*<0.001). The brain water content in BDL + CMN (78.27 ± 0.31) had a considerable decrease compared with BDL and vehicle (*P*<0.001).

***Permeability of the blood-brain barrier***

 Evans blue content in all groups 72 hr after induction of extrahepatic and intrahepatic cholestasis is shown in Figure 2. Figure 2-A shows that the quantity of Evans blue dye in APAP (15.2 ± 1.19) increased significantly compared with the sham group (6 ± 0.48) (*P*<0.001). Evans blue content in the APAP + CMN (11 ± 0.36) showed a significant decrease compared with APAP and vehicle (14.55 ± 0.66) groups (*P*<0.001). On the other hand, no significant difference was observed in the Evans blue content between APAP and vehicle. Figure 2-B shows that Evans blue content in BDL (7.83 ± 1.72) showed no significant difference compared with the sham group.

***ICP*** ***measurement***

 Figure 3 shows ICP changes in all groups at different times. Hepatic injury causes an increase in ICP at 0, 24, 48, and 72 hr after extrahepatic and intrahepatic damage. ICP showed a significant increase in all groups at all times compared with the sham (5 ± 0.8) group (Figure 3-A). No significant difference was observed in ICP between APAP and vehicle. Figure 3-B shows that ICP in BDL + CMN had a significant decrease compared with BDL and Vehicle at 0, 24, 48, and 72 hr after induction of cholestasis (*P*<0.001). ICP had no significant difference between BDL and vehicle. 

***Measurement of serum ammonia***

Biochemical results obtained from the measurement of serum ammonia are shown in Figure 4. Figure 4-A shows a sharp increase in serum ammonia levels in APAP (0.7 µm/l ± 1.02), which indicated hepatic damage and was significantly different from the sham (0.3 µm/l ± 0.51) group (*P*<0.001). 

The amount of serum ammonia in CMN + APAP was significantly decreased compared with APAP and vehicle (*P*<0.001). On the other hand, serum ammonia levels were not significantly different in APAP and vehicle (0.7 µm/l ± 1.02). In Figure 4-B, serum ammonia levels in the BDL group showed a significant increase compared with the sham (0.3 µm/l ± 0.51) group (*P*<0.001).

Furthermore, serum ammonia levels in BDL + CMN (0.8 µm/l ± 1.12) showed a significant decrease compared with BDL and vehicle (*P*<0.001), while no difference was observed between serum ammonia in BDL and vehicle.

***The analysis of liver histopathology***

The BDL and APAP effect on hepatocellular morphology was observed in H&E staining of the brain sections. Our results showed no significant pathological changes in the sham group (Figures 1-4, 2-4). BDL and APAP groups had severe inflammatory cell infiltration. Pretreatment with CMN significantly preserved inflammatory infiltration in these groups (Figures 1-4, 2-4).  

**Figure 1:**
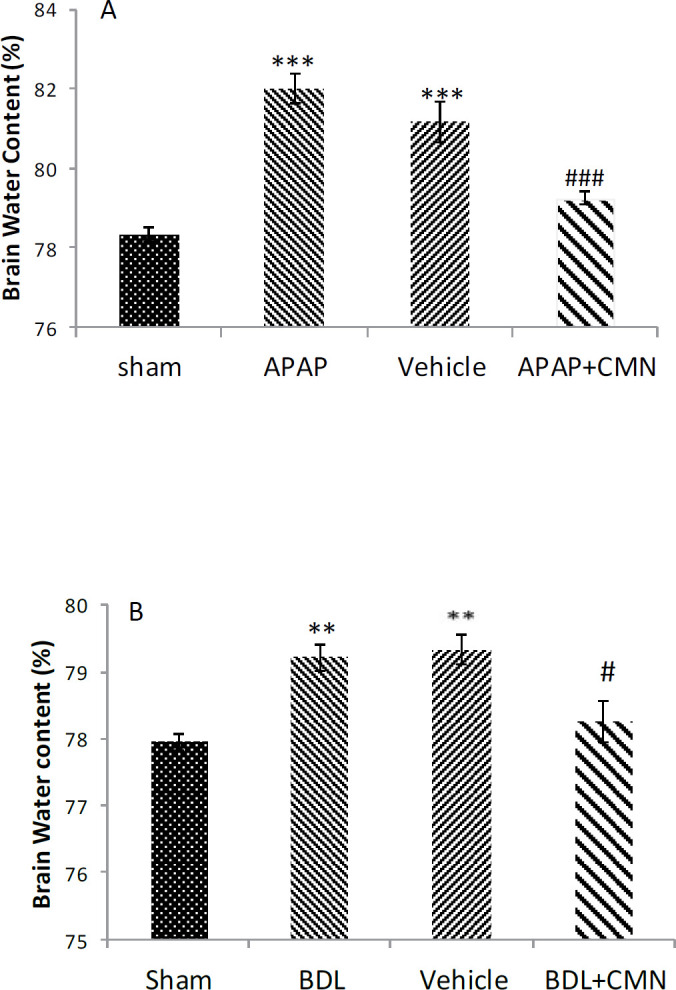
Percentage of brain water content caused by intrahepatic or extrahepatic damage in rats (n = 6 per group). Results were reported as mean ± SEM. Figure A-1: *P*<0.001 ***: significant difference between APAP and sham group. *P*<0.001 ###: significant difference between APAP+CMN and APAP. Figure B-1: *P*<0.01 **: significant difference between BDL and sham. *P*<0.05 # significant difference between BDL+CMN and BDL

APAP: Acetaminophen; CMN: Curcumin; BDL: Bile duct ligation

**Figure 2:**
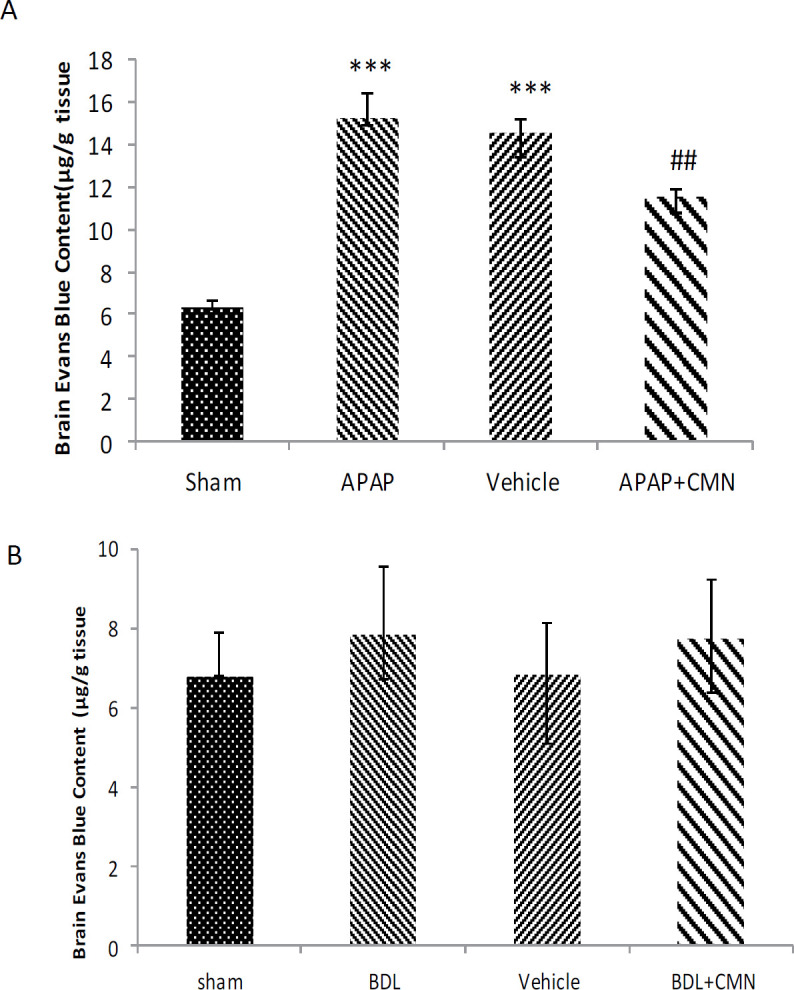
Evans blue content (μg/g tissue) following the induction of extrahepatic and intrahepatic cholestasis in rats (n=6 per group). The results were reported as mean±SEM

*P*<0.001 ***: significant difference between APAP and sham group. *P*<0.01 ##: significant difference between APAP+CMN and APAP

APAP: Acetaminophen; CMN: Curcumin; BDL: Bile duct ligation

**Figure 3:**
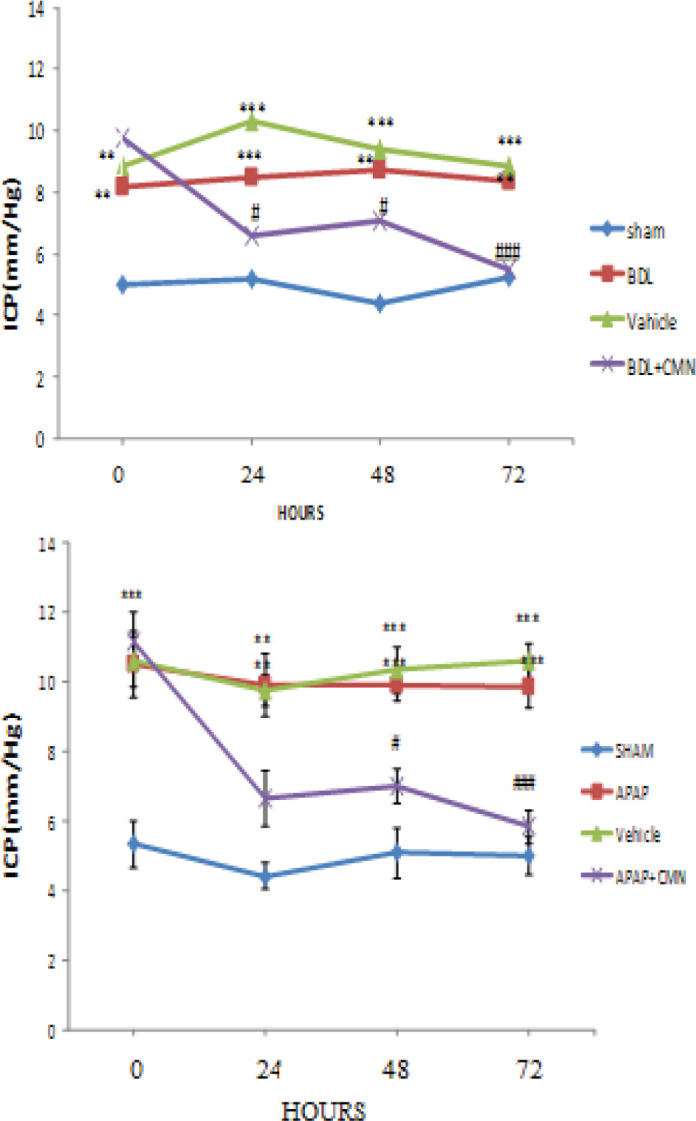
Changes in ICP (mmHg) following the induction of extrahepatic and intrahepatic cholestasis in rats (n=6 per group). The results were reported as mean±SEM. *P*<0.001 *** and *P*<0.01 **: significant difference between APAP and sham group. *P*<0.001 ### *P*<0.05, #: significant difference between CMN+APAP and APAP group. *P*<0.001. ### *P*<0.05, #: significant difference between BDL+CMN and BDL

APAP: Acetaminophen; CMN: Curcumin; BDL: Bile duct ligation

**Figure 4:**
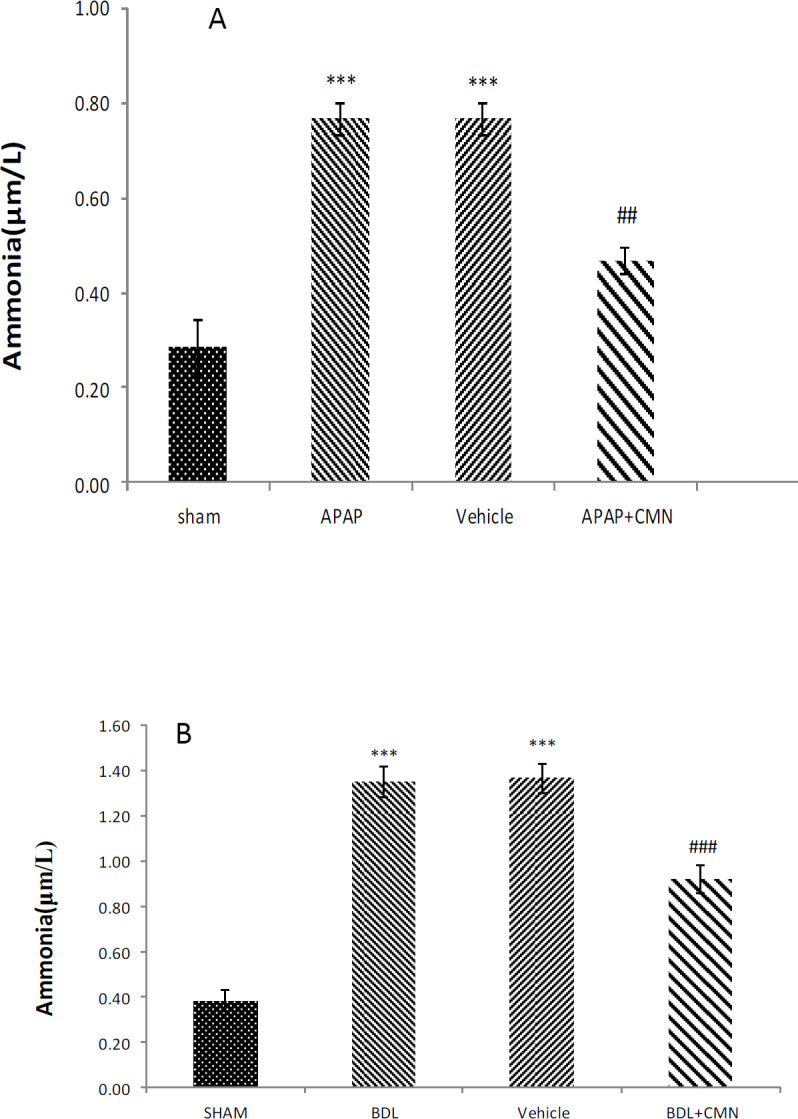
Serum ammonia levels (μm/l) following induction of extrahepatic and intrahepatic cholestasis in rats (n=6 per group). The results were reported as mean±SEM. *P*<0.001 ***: significant difference between APAP and sham group. *P*<0.01 ##: significant difference between CMN+APAP and APAP. *P*<0.001 *** significant difference between BDL and vehicle and sham groups. *P*<0.001 ### significant difference between BDL+CMN and BDL

APAP: Acetaminophen; CMN: Curcumin; BDL: Bile duct ligation

**Figure 1-4:**
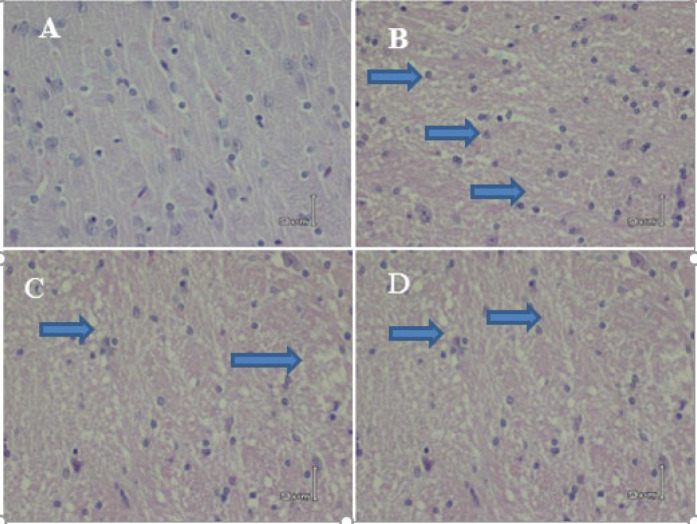
Comparison of stained brain tissue edema with H & E (40 ×) in different groups, A (Sham), B (APAP) severe edema, C (Vehicle), and D (CMN + APAP) mild edema

**Figure 2-4:**
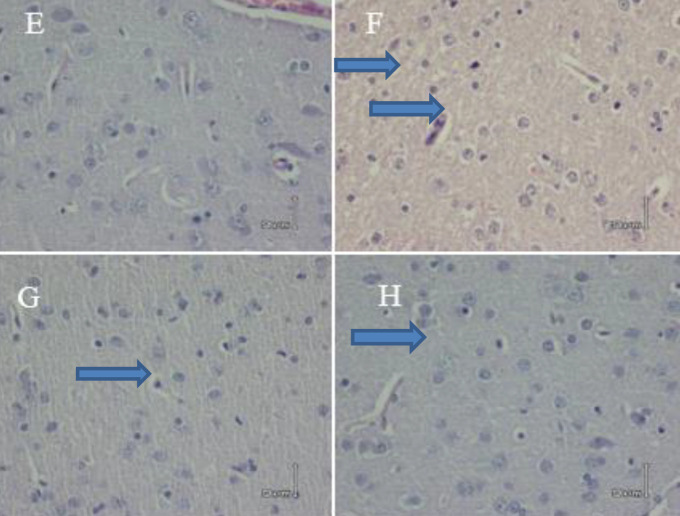
Comparison of stained brain tissue edema with H & E (40 ×) in different groups, E (Sham), F (BDL) severe edema, G (Vehicle), and H (CMN + BDL (mild edema)

## Discussion

For the first time in this study, curcumin’s protective effects and some of its possible mechanisms were investigated in the animal model of HE. The results of this study showed that curcumin decreases brain edema, BBB permeability, ICP, and serum ammonia in animals with HE. The liver is the main organ of metabolism and detoxification in the body. HE is a neuropsychological disorder that is seen in patients with liver failure (1).

Like alcoholic encephalopathy and other encephalopathies, extrahepatic cholestasis creates encephalopathy via neurotoxin deposition in the brain (23). Some hepatic injuries lead to HE in 60–80% of the patients. Brain edema, along with increased ICP and cerebral herniation, is the most common cause of death in patients with HE (24).

HHE appears to be associated with toxic products produced by intestinal bacteria such as ammonia, mercaptans, short-chain fatty acids, and phenols. It should be naturally metabolized or excreted quickly. In liver damage, damaged hepatocytes cannot detoxify these substances. Therefore, these drugs enter the brain and alter its function (25, 26). Failure in liver cells decreases the liver’s ability to neutralize ammonia and remove it (27). 

Hyperammonemia is considered an important factor in cerebral and neurological changes. By changing neurotransmitters’ metabolism and inducing neuronal toxicity, hyperammonemia plays a significant role in the pathogenesis of HE (27). This study showed that curcumin decreased the serum ammonia levels in the liver of animals with HE. Curcumin has been reported as a potent iron chelator, which prevents abnormalities in the liver and spleen by preventing iron overload (28). Mehmet Tokaç *et al*. (2013) demonstrated that curcumin plays a hepatoprotective role by improving liver function parameters and decreasing the levels of MDA and NO, along with increasing the levels of antioxidants (29). Another study showed that curcumin might be useful for health by modulating lipid metabolism in alleviating hepatosteatosis and suppressing atherogenesis (30). As a result, it can substantially affect hepatic detoxification capacity, which is confirmed by decrease in ammonium serum concentrations in this study. Shapiro *et al*. (2006) showed that a high dose of curcumin decreases liver enzyme levels and blood ammonia to a greater extent after thioacetamide-induced hepatotoxicity, which is consistent with the results of this study (31). 

The results of this study also showed that curcumin could lead to a decrease in the ICP after intrahepatic and extrahepatic damages, the maximum decline was observed 72 hr after curcumin administration. A study (2012) reported that 20–30% of mortality in patients with acute liver failure is due to increased ICP and reduced cerebral perfusion pressure (32). Furthermore, the production of pro-inflammatory cytokines in the brain is associated with uncontrolled ICP(33). Studies have shown that curcumin plays an essential role in reducing vascular inflammation and acute injury caused by TBI (34). 

Curcumin crosses the BBB and applies its neuroprotective effects on the damaged neurovascular network (35). Other studies have shown that clinically achievable doses of curcumin attenuated cerebral edema development via reducing neuroinflammatory activation and attenuated expression of the glial water channel, AQP4 (36). 

Curcumin inhibits lipid peroxidation in the brain via its iron-chelating role and the consequent reduction of Fe (II) in the brain (37). 

Neural inflammation breaks down the BBB, allows the blood cells to exit from the bloodstream, and creates a cascade of secondary brain damages (38). Morphological change, such as cell swelling in astroglial cells, is another characteristic of HE (39). 

The decrease in the BBB permeability and cerebral edema in encephalopathy were the other effects of curcumin in this study as curcumin’s protective effects were apparent in histopathological observations. In confirming these effects, Qi *et al*. (2017) showed that curcumin decreases BBB permeability and brain edema in the face of chronic hydrocephalus after intraventricular hemorrhage (IVH) via maintaining the overall structure of the BBB (40). Several reports have shown the critical role of curcumin in improving permeability and reducing initial neuronal damage in ischemic attacks (41) and intracranial hemorrhage (42).

It has also been found that use of curcumin after subarachnoid hemorrhage decreased brain water content, BBB damage, and neurological problems (43). A different study showed that curcumin reduced cerebral edema and improved BBB disorder in rats after subarachnoid hemorrhage. This process is due to a decrease in the expression of VEGF and MMP9 and the increased expression of claudin, occludin, and ZO-1 (44). Another study showed that curcumin could decrease the expression of AQP4 levels in the brain and reduce brain edema (45). However, in this study that evaluated the protective effect of curcumin on encephalopathy caused by intrahepatic and extrahepatic damage in male rats, the presented parameters are insufficient. This is one of the limitations of the present study. It is recommended that further research focus on assessing anti-inflammatory and antiapoptotic evaluations after encephalopathy.

## Conclusion

The findings of this study suggest that curcumin has protective effects on encephalopathy caused by extrahepatic and intrahepatic damages. Considering the protective effects of curcumin on HE, it is recommended to investigate its action mechanism in future studies by examining its anti-inflammatory and anti-oxidant effects.
